# Perturbations during Gait: A Systematic Review of Methodologies and Outcomes

**DOI:** 10.3390/s22155927

**Published:** 2022-08-08

**Authors:** Zoe Taylor, Gregory S. Walsh, Hannah Hawkins, Mario Inacio, Patrick Esser

**Affiliations:** 1 Centre for Movement, Occupation and Rehabilitation Sciences, Oxford Brookes University, Oxford OX3 0BP, UK; 2 Department of Sport Health Sciences and Social Work, Oxford Brookes University, Oxford OX3 0BP, UK; 3 Research Centre in Sport Sciences, Health Sciences and Human Development, University Institute of Maia, 4475-690 Maia, Portugal

**Keywords:** gait, perturbation, balance, falls

## Abstract

Background: Despite extensive literature regarding laboratory-based balance perturbations, there is no up-to-date systematic review of methods. This systematic review aimed to assess current perturbation methods and outcome variables used to report participant biomechanical responses during walking. Methods: Web of Science, CINAHL, and PubMed online databases were searched, for records from 2015, the last search was on 30th of May 2022. Studies were included where participants were 18+ years, with or without clinical conditions, conducted in non-hospital settings. Reviews were excluded. Participant descriptive, perturbation method, outcome variables and results were extracted and summarised. Bias was assessed using the Appraisal tool for Cross-sectional Studies risk of bias assessment tool. Qualitative analysis was performed as the review aimed to investigate methods used to apply perturbations. Results: 644 records were identified and 33 studies were included, totaling 779 participants. The most frequent method of balance perturbation during gait was by means of a treadmill translation. The most frequent outcome variable collected was participant step width, closely followed by step length. Most studies reported at least one spatiotemporal outcome variable. All included studies showed some risk of bias, generally related to reporting of sampling approaches. Large variations in perturbation type, duration and intensity and outcome variables were reported. Conclusions: This review shows the wide variety of published laboratory perturbation methods. Moreover, it demonstrates the significant impact on outcome measures of a study based on the type of perturbation used. Registration: PROSPERO ID: CRD42020211876.

## 1. Introduction

Balance capacity assessment is often performed with the use of perturbation testing [1]. Assessing balance is key in fall risk assessment, particularly among older adults (OAs) over the age of 65 years, with 30% of OAs and 50% of those over the age of 80 years experiencing a fall every year [2]. Fall risk has a close association to balance capacity; as a result, in recent years, researchers have developed a variety of methods of perturbing participants to understand balance and recovery mechanisms [3,4,5]. These perturbations have taken many forms including visual field oscillations and pulls and pushes applied to the waist and floor level translations [6,7,8]. An individual’s ability to produce an appropriate mechanical response to these perturbations can reduce fall risk [5,9].

In the context of this review, a balance perturbation refers to an external action applied to a participant, causing disruption to the balance control [10]. Various approaches have been developed to recreate a balance perturbation and assess balance responses in a safe, controlled environment. This is achieved by having controlled mechanical perturbation delivery applied to participants to aid in the clinical investigation of risk factors to future fallers [1]. As a result, this review collected spatiotemporal, kinetic, kinematic, and muscle activation outcome variables, all of which are used in reporting participants’ responses to balance perturbations.

Previous reviews have focused on the effectiveness of perturbation training in reducing fall frequency or improving balance perturbation response amongst OAs and clinical populations [11,12,13]. Although a previous review [14,15] has identified significant diversity in perturbation methods, there is currently no exhaustive analysis of current mechanical perturbation methods used and their corresponding biomechanical responses. This warrants investigation, as the interpretation of perturbation responses is commonly used to assess balance performance fall risk in a range of populations. However, perturbations applied in different ways will result in diverse mechanical responses in participants and will reflect different real-world slip, trip, and fall scenarios. Therefore, understanding common perturbation techniques, the outcome measurements made, and the corresponding biomechanical outcomes are important for research intending to utilise perturbation paradigms to investigate balance control. Therefore, this review aims to identify and describe the current methods and outcome variables used to apply and report participants’ responses to balance perturbations during gait across all adult populations.

## 2. Materials and Methods

This review is registered in the PROSPERO Register of Systematic Reviews (reference number CRD42020211876) and was reported according to the PRISMA2020 guidelines [16]. A deviation from the PROSPERO registered protocol was made in the reported systematic review, whereby the age of included participants was lowered to include all adults aged 18 years or older, instead of focusing on those aged >65 years. This change was made to ensure that the range of current perturbation methods and outcome measures were captured in the review.

Search strategy: Web of Science, CINAHL, and PubMed online databases were searched using the following search string: (fall * OR perturb * OR slip * OR trip *) AND (adult *) AND (mediolateral OR medio lateral OR medio-lateral OR frontal plane) AND (gait OR walk * OR locomotion). The same search string was used in each database. The final search was conducted on 30th May 2022. Once duplicates were removed, remaining article titles and abstracts were screened against the eligibility criteria. Full texts of the remaining titles were retrieved and screened against the eligibility criteria. At each stage of screening, all articles were screened by two reviewers, agreement was >95%, and any discrepancies were resolved by a third reviewer.

Eligibility criteria: The inclusion criteria included (1) participants aged 18 years or older, (2) participants were subjected to mechanical balance perturbation during gait, (3) outcome variables reported included kinetic, kinematic, spatiotemporal, or muscle activation, (4) peer-reviewed articles published during or after 2015, and (5) published in English. Exclusion criteria included (1) balance perturbations did not occur during continuous gait, e.g., standing, at gait initiation, during a single stepping task, reaching, or a specific sporting scenario; (2) there was no perturbation reported; (3) observational studies, case studies, or literature reviews.

Risk of bias: The Appraisal tool for Cross-sectional Studies (AXIS) risk of bias assessment tool was used to assess potential risks of bias in the methods and outcome variables reported [17]. All included studies were assessed using 20 questions within the categories of introduction, methods, results, discussion, and other.

Data extraction: Data synthesis was limited to a qualitative evaluation of the balance perturbation methods and outcome variables. Participant data extracted included demographic parameters (number, age, sex, height, body mass, body mass index, and fall history) and any stated comorbidities. Method specific variables extracted included locomotion type (e.g., treadmill or overground walking and walk speed), balance perturbation type and details (timing within gait cycle, direction, acceleration, frequency, force, intensity, mode of application) balance perturbation acclimation procedures and gait outcome variables.

## 3. Results

**Figure 1:**
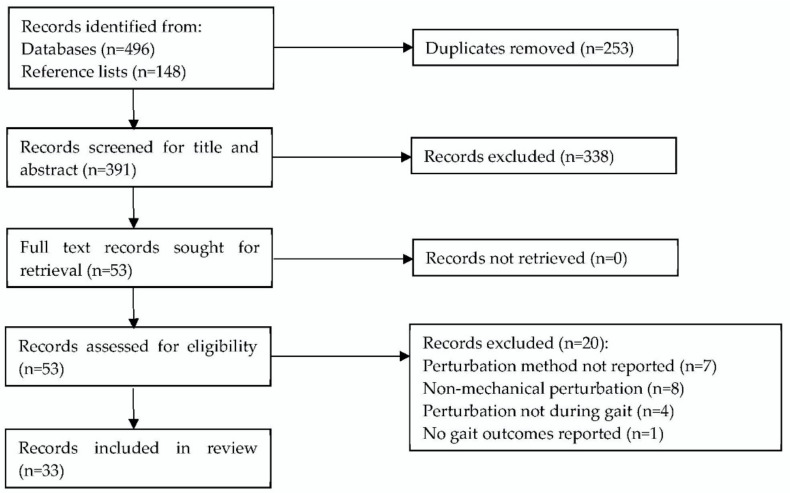
PRISMA flow diagram of search and exclusion criteria [16].

Searches of Web of Science, CINAHL, and PubMed online databases produced 496 records, with a further 148 records, including duplicates, identified from screening included records’ reference lists. This was reduced to 391 records following duplicate removal. A screening of search results established 33 studies that met the inclusion criteria. A PRISMA flow diagram of the study selection is given in Figure 1.

Risk of bias: A lack of detail regarding participant recruitment methods was common. All included studies clearly stated the aim and selected appropriate methods and outcome measures for the aims. Overall, there was a low risk of bias across all studies. The full results of the AXIS risk of bias assessment are given in Appendix A Table A1.

Characteristics of included studies: All included studies were laboratory based: 19 used a treadmill and 14 perturbed participants during overground walking. A total of 779 participants from 33 studies were included (Table 1). 

Balance perturbation methods and details: The full details of balance perturbation methods of each paper are given in Table 2. Eight studies used a split-belt treadmill [8,18,19,20,21,22,23,24], five used a single-belt treadmill [9,25,26,27,28], and six studies oscillated the treadmill during gait [29,30,31,32,33,34].

Eight studies used participant-worn devices to perturb participants, five were waist- or hip-worn attachments connected to external structures which pulled or pushed participants [7,35,36,37,38]. One device applied vibrations to the hip abductor muscles [39]. One study used an oscillating pendulum system worn on participants’ backs [40], one study applied a mechanised block to the foot [41], and one study applied a narrow treadmill-based stepping task while participants were executing a cognitive task [42].

In the context of this review, a translational platform refers to a movable floor level platform designed to cause perturbation to participants during gait. Three studies perturbed participants during overground walking using a translational platform [43,44,45], one induced a perturbation by oiling the floor [46], and one used a physical therapist who applied the balance perturbations at waist level [47].

**Table 1:** Summary of included studies’ participant details.

Reference	Sample Size	Population	Age Years (SD)	Height m (SD)	Body Mass kg (SD)
Aprigliano et al. [20]	5	Healthy YA	25.4 (3.1)	1.7 (0.1)	63.2 (11)
Aprigliano et al. [18]	15	Healthy YA	26.1 (1.3)	1.78 (0.06)	68.8 (12.3)
Aprigliano et al. [19]	6	Healthy OA	68.7 (5.2)	1.76 (0.1)	76.9 (7.9)
Arvin et al. [39]	12	Healthy YA	27.3 (1.7)	1.68 (0.11)	60.6 (10.5)
	18	Healthy OA	70.8 (6.8)	1.70 (0.86)	80.1 (8.8)
Best et al. [40]	12	Healthy YA	21.8 (1.0)	1.802 (0.092)	72.3 ((11.2)
Capin et al. [47]	40	Post-operative ACL rupture	23.0 (7.0)	1.78 (0.07)	86.0 (11.5)
Francis et al. [42]	12	Healthy YA	23.6 (3.9)	1.69 (0.25)	70.7 (11.3)
	11	Healthy OA	71.2 (4.2)	1.64 (0.06)	66.9 (9.6)
Golyski et al. [24]	10	Healthy YA	24 (3)	1.76 (0.11)	74.1 (12.0)
Haarman et al. [25]	10	Paretic OA	52 (16)	1.75 (0.06)	82.5 (13.6)
Hof and Duysens [38]	9	Healthy YA	19–23	Not reported	Not reported
Kao et al. [31]	18	Healthy YA	20.4 (1.5)	1.72 (0.10)	69.2 (11.7)
Kreter et al. [41]	5	Healthy YA	24 (5)	Not reported	Not reported
	5	Concussed YA	25 (5)		
Kurz et al. [9]	53	Healthy OA	80.1 (5.6)	1.58 (0.09)	68.3 (13.9)
Lee-Confer et al. [46]	16	Healthy YA	21–35	Not reported	Not reported
Madehkhaksar et al. [26]	10	Healthy YA	26.4 (4.1)	1.7 (0.08)	64.4 (12.5)
Martelli et al. [35]	8	Healthy YA	29.9 (4.9)	1.68 (0.08)	67.9 (9.4)
Martelli et al. [7]	18	Healthy YA	23.9 (4.2)	1.75 (0.08)	70.3 (8.1)
McIntosh et al. [43]	11	Healthy YA	23.8 (3.1)	1.76 (0.01)	71.2 (12.4)
	10	Healthy OA	71.1 (3.1)	1.68 (0.01)	72.6 (11.1)
Nestico et al. [32]	16	Healthy YA	20–35	Not reported	Not reported
Onushko et al. [27]	15	Healthy YA	21.3 (1.4)	1.7 (0.1)	68.8 (10.7)
Punt et al. [22]	38	Stroke Survivors OA	60.2 (9.5)	1.72 (0.12)	85 (19.6)
Rieger et al. [21]	30	Healthy OA	70.1 (4.5)	1.72 (0.09)	75.2 (10.2)
Roeles et al. [28]	9	Healthy YA	25.1 (3.4)	1.76 (0.09)	76.6 (15.1)
	9	Healthy OA	70.1 (8.1)	1.70 (0.11)	77.9 (10.5)
Rosenblum et al., 2020 [29]	12	Healthy YA	26.9 (3.4)	1.68 (0.07)	63.67 (10.26)
	12	Healthy OA	69.5 (5.2)	1.70 (0.07)	78.34 (16.22)
Rosenblum et al. [33]	20	Healthy YA	27 (3)	1.67 (0.08)	62.5 (10.7)
Rutherford et al. [45]	32	Knee	61 (6)	1.69 (0.10)	85.5 (14.3)
		Osteoarthritis OA			
	44	Healthy OA	60 (6)	1.68 (0.08)	70.7 (13.0)
Sheehan et al. [30]	22	Healthy YA	27.2 (6.9)	1.76 (0.095)	84.8 (13.9)
Shulman et al. [44]	18	Healthy YA	21.7 (2.6)	1.80 (0.1)	72.8 (11.0)
	16	Healthy OA	75.6 (5.3)	1.70 (0.1)	72.7 (13.4)
Taborri et al. [34]	12	Healthy YA	26 (3)	1.71 (0.05)	64.9 (9.6)
van Hal et al. [23]	20	Not reported	Not reported	Not reported	Not reported
Vervoort et al. [8]	75	Healthy YA and OA	48.1 (17.95)	1.75 (0.10)	73.81 (10.81)
Vlutters et al. [36]	10	Healthy YA	25 (2)	1.8 (0.11)	67 (12)
Zadravec et al. [37]	7	Healthy YA	33.4 (8.5)	1.81 (0.05)	80.1 (11.6)

ACL: anterior cruciate ligament, OA: older adult, YA: younger adult.

The largest perturbation displacement described was 18.0 cm, and this was delivered by two studies using medio-lateral (ML) treadmill translation and ML translation of a platform during overground gait [9,44]; the smallest displacement reported was 1.0 cm by ML treadmill translation [9]. The largest acceleration reported was 16.0 m s^−2^ by ML treadmill translation [9], and the smallest was 0.1613 m s^−2^ with a split belt treadmill [21]. Where a perturbation number was reported, the average number of perturbations applied to each participant was 14, with all studies reporting at least 10 perturbations per participant.

Timing of balance perturbation onset: Fourteen studies initiated balance perturbations at heel strike [7,18,19,20,21,22,23,26,27,28,29,35,37,43]. Six studies applied balance perturbations continuously or at intervals during a walking trial [8,9,30,31,40,42]. Two studies perturbed participants at toe-off [25,36], one study during mid-stance [45], one study during single or double support [33], and one study prior to toe-off [44]. One study applied perturbations at multiple points throughout the gait cycle [38]. One study perturbed participants at 40% of stance phase [39], and another study perturbed participants at 10, 15, 20, and 30% of the gait cycle [24]. Three studies did not specify when within the gait cycle perturbations were applied [32,41,47].

Preferred or prescribed walking speed: Eighteen studies prescribed a fixed walking speed when subjected to perturbations [7,8,21,22,24,25,26,29,30,31,34,35,36,38,40,42,43,46]. Twelve studies allowed participants to walk at their preferred walking speed when perturbed [9,18,19,20,23,27,28,32,33,39,44,45]. Three studies did not report if gait speed was prescribed or self-selected [37,41,47].

**Table 2:** Details of perturbation methods.

Reference	Perturbation Cause	Perturbation Details
Aprigliano et al. [20]	Split belt TM	Initiation at HS, 5 left belt perturbations 5 right belt
Aprigliano et al. [18]	Split belt TM	Initiation at HS, 5 left belt perturbations 5 right belt
Aprigliano et al. [19]	Split belt TM	Initiation at HS, anterior, or lateral translation of right belt
Arvin et al. [39]	Hip abductor vibration during gait	Mean vibration duration 0.41 s, 40% stance phase
Best et al. [40]	Pendula mass oscillating during gait	Pendula mass 6.4% of body mass, frequency at 130%, and 70% of strides
Capin et al. [47]	OG walking physical therapist perturbations	Lack of detail, progressive perturbation applied by trained physical therapist
Francis et al. [42]	Narrow TM walking, cognitive task	Perturbation was sum of sinusoids waves to the virtual hallway, amplitude 0.175 m
Golyski et al. [24]	Split belt TM	Initiation at 10, 15, 20, and 30% of gait cycle, individual belt acceleration then deceleration for 30% gait cycle duration, 10 each leg at each initiation point
Haarman et al. [25]	Movable TM	Initiation at toe-off, 150 ms block pulses equal to 4, 8, 12, and 16% of body mass
Hof and Duysens [38]	Waist belt, right side, pneumatics	Push and pulls of 100 ms throughout gait, at 2.7–12.4 kg m s^−1^
Kao et al. [31]	Movable TM	Continuous pseudo-random amplitude ML translations with max amplitude 0.05 m for 150 s
Kreter et al. [41]	OG walking mechanised shoe	5–12° ankle eversion randomly between 3rd and 6th stride of a 7.5 m walk
Kurz et al. [9]	Movable TM	Perturbation every 20–40 s for 14 min, 0.01–0.18 m, 0.5–16.0 m s^−2^
Lee-Confer et al. [46]	OG low-friction floor	Floor surface was coated in mineral oil to induce slip
Madehkhaksar et al. [26]	Single belt TM	16 perturbations of 2.5 m s^−1^, HS within four five min blocks
Martelli et al. [35]	Waist belt, cable pulleys	Pulsation rise, hold, fall time of 150 ms. 10, 15, 20% body mass, HS
Martelli et al. [7]	Waist belt, cable pulleys	Pulsation rise, hold, fall time of 150 ms. 10, 15, 20% BM, HS
McIntosh et al. [43]	OG walking floor translation	0.15 m, 0.316 ms 2.4 m s^−2^, HS
Nestico et al. [32]	ML TM oscillations	ML translation 200 ms acceleration followed by 200 ms deceleration of 0.072 m at 1.8 m s^−2^
Onushko et al. [27]	Single belt TM	ML ±0.08, 0.165, and 0.25 m oscillations initiated at HS
Punt et al. [22]	Split belt TM	16 ML perturbations of 0.045 m separated by 15 s
Rieger et al. [21]	Split belt TM	0.31 s, 0.05 m, 0.1613 m s^−2^, HS medial translation
Roeles et al. [28]	Single belt TM	0.7 s, 0.05 m, 2.04 m s^−2^
Rosenblum et al. [29]	ML TM oscillations	0.15 m displacement in 0.92 s, initiated at HS
Rosenblum et al. [33]	ML TM oscillations	ML translation of 0.15 m for 0.92 s at single or double support
Rutherford et al. [45]	OG walking ML floor translation	ML translation of 0.01 or 0.03 m during mid-stance at 0.1 m s^−1^
Sheehan et al. [30]	ML TM oscillations	Continuous 3 min ± 12 cm displacement from centre
Shulman et al. [44]	OG walking ML floor translation	6 left, 4 right side prior to toe-off at 0.6 ms, 2 m s^−2^, 0.18 m
Taborri et al. [34]	Movable TM	AP acceleration at 0.5 m s^−2^ or ML translation of 0.16 m at 0.18 m s^−2^
van Hal et al. [23]	Split belt TM	Initiation at HS, lateral pull, 0.66 m s^2^ of 0.05 m
Vervoort et al. [8]	Split belt TM	Randomly, one belt at 0.7 m s^−1^, one at 1.4 m s^−1^
Vlutters et al. [36]	Waist belt, motors	150 ms block pulses, forces 4, 8, 12, 16% BM
Zadravec et al. [37]	Waist belt	5 repeats of 4 directions. 15% BM, 150 ms

BM: body mass, HS: heel strike, AP: anterior-posterior, ML: medio-lateral, OG: overground, TM: treadmill.

Outcome variables: Full details of the outcome variables reported by each study are given in Table 3 along with a summary of the key results. A wide range of kinetic, kinematic, spatiotemporal, and muscle activity outcome variables were reported.

Summary of Kinetics: Two studies reported differing vertical ground reaction force (vGRF) results: one reported an increase [36] and the other reported no significant effect of split belt perturbation on participants’ vGRF in normal gait [22]. Perturbation exposure increased angular momentum range and increased variability of angular momentum [30]. Stride to stride variability increased as did range of angular momentum [30]. Varying responses to perturbation were found for joint moments, and manually applied perturbations did not alter hip, knee, and ankle joint moments [47]; however, sagittal and frontal knee moments were increased by ML surface translations [45].

Summary of Kinematics: Eight studies reported results for joint angles, excursions, or RoM [18,19,20,31,45,46,47]; effects with a tendency to increase flexion, RoM, and the variability of lower limb joints were reported in all studies. ML foot placement was reported by one study [39], with three others reporting combined anterior-posterior (AP) and ML foot placement [25,36,37].

Seven studies reported CoM displacements and CoM velocity [23,26,36,39,40,43,44]. CoM sway velocity, sway area, and mean sway during gait was reported by one study [9]. Five studies reported using CoP displacement as an outcome variable [8,23,36,39,44]. Seven studies reported on the margin of stability (MoS) [7,20,22,24,28,33,40] and one reported on the base of support (BoS) area [7]. All studies reported an increase in MoS; however, one study reported a decrease in MoS [20].

A linear relationship was identified between CoM velocity, CoP, and GRF during perturbation recovery [36]. Conflicting findings were reported for CoM velocity with overground platform translation increasing CoM velocity [43], while hip abductor vibration showed no effect on CoM velocity [39]. On the contrary, overground platform translation perturbation showed no effect on ML CoM displacement or normalised ML displacement [43]. AP and ML treadmill translation significantly decreased CoM AP sway [9].

Six studies reported measures of dynamic stability [19,26,28,31,35,42]. AP and ML treadmill belt translations of 2.5 m s^−1^ [26], AP and ML waist belt pulls and pushes [35] and medial belt translation [21], and narrowed step width [42] all reportedly increased participants’ dynamic stability. Two studies reported decreased dynamic stability in response to perturbation [19,31].

Summary of Spatiotemporal: Eight studies reported the duration of gait phases [18,19,20,22,25,36,38,41]. Conflicting findings were reported in most spatiotemporal variables. ML treadmill translation caused a decrease in participants’ step time [27], while the same perturbation method by a different study showed no effect of the perturbation on step time [22]. However, overground floor translations showed a decrease in swing time [44]. Inconsistent results were also found regarding single support time; ML treadmill oscillations [25] and medial treadmill translations [21] increased single support time. However, anterior and lateral split belt treadmill translations [19], hip abductor muscle vibration [39], and differing split belt speeds [8] all showed decreased single support time. Conversely, regarding double support duration, all studies reported a decreased duration following the perturbations of anterior and lateral split belt treadmill translations [19], hip abductor muscle vibration [39], mechanised shoes [41], and differing split belt speeds [8]. AP and ML treadmill translations were shown to increase participants’ foot contact time; however, this was not specific to single, double, or both support durations [9].

Included studies reported contradicting results of participants’ step width, showing an increase following ML treadmill translation [27,32], AP and ML treadmill translations [26], single belt AP and ML treadmill translation [28], split belt treadmill accelerations [24], and hip abductor muscle vibration [39] during perturbed gait. Contradicting this, anterior and lateral split belt treadmill translations [19], gait post vibration of hip abductor muscles perturbation [39], and medial treadmill translations [21,31] were all showed to reduce step width. Further opposing results showed no significant effect on step width when participants performed a narrow stepping task [42] or were subjected to the oscillating mass perturbation [40], overground AP and ML floor translations [43], and ML treadmill translations [22]. Only one study reported step width variability and found no significant effect of a narrow walking task [42].

Further contradicting results were seen in step length findings, with AP and ML waist pulls and pushes [35] and medial treadmill belt translations [21] increasing step length. Opposing this were findings that treadmill perturbations of 0.05 m at 2.04 m s^−2^ decreased step length [28], as did ML treadmill translations [27] and AP and ML treadmill translations of 2.5 m s^−1^ [26]. Just one study described that an age effect of increased age also reduced step length in response to different split belt speeds [8]. Finally, there was no significant difference in step length following overground AP and ML floor translations [43], ML treadmill translations [22], or a narrow stepping task [42].

All studies reporting step frequency were agreed to a significant increase following perturbation by treadmill translation [26,28] or split belt accelerations [8]. Additional findings showed AP perturbations to reduce stride duration significantly more compared with ML or diagonal perturbations [18,20].

Summary of Muscle activations: Four studies reported muscle activations [25,33,38,45]. Gluteus medius activation increased significantly as a result of perturbation application [25]. The tibialis anterior, peroneus longus, and soleus activity were all reported to increase in response to gait perturbation [38], as did mean spectrum power [33]. However, the magnitude of activity in the first principal components of quadriceps, hamstrings, and gastrocnemius activity decreased following overground floor translations [45].

**Table 3:** Outcome measures and the perturbation effect.

Reference	Outcome Variables	Summary of Change in Outcome Variables
Aprigliano et al. [20]	Hip, knee, ankle joint angles, compensatory time, MoS	Joint angles altered, no detail. Increased compensatory time with perturbation intensity. Decreased MoS.
Aprigliano et al. [18]	Stride time, stance %, hip, knee, ankle joint angles.	Decreased stride time, stance %, hip, knee, thigh, shank, foot RoM. Increased ankle RoM.
Aprigliano et al. [19]	Dynamic stability, step width, single and double support time, hip, knee, ankle RoM. MoS.	Decreased dynamic stability, step width, single and double support time, knee, ankle RoM. Increased frontal plane hip RoM, sagittal and frontal plane MoS. No change sagittal plane hip RoM.
Arvin et al. [39]	CoM position, CoM velocity, step width.	Decreased step width at perturbation, increased step width post perturbation. No change CoM position or velocity.
Best et al. [40]	MoS, CoM amplitude, step width, step length, step time.	Increased MoS only in out of phase. Decreased CoM amplitude. No change step width, step length, step time.
Capin et al. [47]	Hip, knee and ankle joint angles, excursions, moment.	Decreased hip, knee, ankle excursions at loading. No change hip, knee, ankle joint angles, moments.
Francis et al. [42]	ML LDE, step width, step length, step width variability, step length variability, gait speed.	Decreased LDE on TM than OG. No change step width, step length, step width variability, step length variability, gait speed.
Golyski et al. [24]	Step width, step length, MoS.	Later perturbation onset increased AP, ML MoS, step width, and step length for perturbed step but lower AP MoS and step length for first recovery step.
Haarman et al. [25]	Gluteus medius EMG activation, CoM velocity, step width, support time, foot velocity, XCoM.	Increased swing time, gluteus medius activation CoM velocity variability. Increased lead foot ML distance from CoM, foot velocity increased. No change step width, step length, XCoM, double support time.
Hof and Duysens [38]	Tibialis anterior, plantar flexor, soleus EMG activation, CoP.	Increased tibialis anterior, plantar flexor, soleus activation. Decreased CoP motion.
Kao et al. [31]	LDE, step width, step length, stride time step width variability, step length variability, MoS, CoM variability, ankle, knee and hip angle variability.	Perturbation decreased AP MoS, step width, length and stride time and increased ML MoS and variability of step width, length and stride time, CoM movement, MoS and all joint angles.
Kreter et al. [41]	Step time, double support time, ML acceleration of foot and trunk.	Perturbation decreased double support time but no effect on step time and increased lateral acceleration of the foot at toe-off and less trunk lateral acceleration in concussed participants but no effect on trunk in control participants.
Kurz et al. [9]	CoM sway, sway velocity, sway area, foot contact time, voluntary stepping.	Decreased CoM AP and ML-sway, sway velocity, sway area. Interaction for foot contact time of the voluntary step execution.
Lee-Confer et al. [46]	Frontal and sagittal plane shoulder angles.	Frontal plane contralateral arm excursion is greater in perturbed than ipsilateral arm and was greater than sagittal plane excursion.
Madehkhaksar et al. [26]	Step length, step width, cadence, dynamic stability, MoS step length, step width, cadence, dynamic stability variability.	Increase step width, cadence, dynamic stability, step length variability, step width variability, cadence variability, dynamic stability variability, AP and ML MoS. Decrease step length.
Martelli et al. [35]	Dynamic stability, MoS, BoS, adaptive response, step length, step width.	Increased dynamic stability, MoS, BoS, adaptive response, step length. Decreased step width.
Martelli et al. [7]	Step length, step width, MoS, BoS.	Increased step length, MoS, step width at first perturbation. Decreased step width overall, AP and ML BoS, AP MoS, at first recovery step. No change at perturbation onset AP BoS, AP and ML MoS.
McIntosh et al. [43]	CoM velocity, CoM displacement, step width, step length, recovery step type.	Increase CoM velocity, CoM displacement range, normalised ML trunk CoM displacement, step width. Decrease step length. 85 of 840 recovery steps were cross-over.
Nestico et al. [32]	Short- and long-term variability (Poincare plots) of step length and width, number of recovery steps required.	Step width and long-term step width variability were greater in perturbed no effect on short term step length variability, no correlation between step width variability and number of recovery steps.
Onushko et al. [27]	Step length, step frequency, step width, MoS.	Increased step frequency, step width, ML MoS, ML MoS variability. Decreased step length.
Punt et al. [22]	Step length, step width, step time, MoS, dynamic stability.	Increase step length, step width, ML MoS, step time. Decrease AP MoS, dynamic stability.
Rieger et al. [21]	Step width, step time, swing time, stance time, step length, dynamic stability, LDE, recovery.	Increase dynamic stability, step time, swing time, stance time, step length, recovery performance. Decrease LDE, step width, recovery time.
Roeles et al. [28]	Step width, step length, step time, MoS.	Increased AP and ML MoS, step width. Decreased step length, step time.
Rosenblum et al. [29]	Step length, step width, recovery time.	Increased step length, step width. Total recovery occurred 4–6 s after perturbation.
Rosenblum et al. [33]	Vastus lateralis and tibialis anterior EMG spectral power, MoS.	MoS, total and individual muscle mean spectral power increased after perturbation.
Rutherford et al. [45]	Knee sagittal and frontal plane moment, knee sagittal RoM, hamstrings, quadriceps and gastrocnemius muscle EMG principal components.	Increased flexion RoM, sagittal and frontal knee moments and decreased muscle activity after perturbation.
Sheehan et al. [30]	Stride variability, angular momentum, gait phases.	Increased stride variability, range of angular momentum. Decreased angular momentum during second double support.
Shulman et al. [44]	BoS, step width, single support time, swing time, step length, gait speed.	Increased BoS, step width. Decreased single support time, swing time, step length. No change gait speed.
Taborri et al. [34]	Sagittal hip, knee and ankle joint range of motion, variability and continuous relative phase coordination.	Increased variability in joint angles, reduced flexion and change in relative phase of all joint combinations.
van Hal et al. [23]	vGRF kinetic, kinematic, spatio-temporal.	Preliminary trial paper, therefore no results.
Vervoort et al. [8]	Cadence, MoS, step length, single and double support time.	Increased cadence, ML MoS of slow leg. Decreased step length, single and double support time.
Vlutters et al. [36]	CoM velocity, CoP, GRF, step length, step width single and double support time.	Linear relationship between CoM velocity, CoP and GRF. Increased vGRF, step length, single and double support time. Decreased step width.
Zadravec et al. [37]	Step time, step width, step length.	Increase step time. Decrease step width, step length. No change in 82% of all stepping response parameters between OG and TM perturbations.

LDE: local divergence exponent, CoM: centre of mass, GRF: ground reaction force, vGRF: vertical ground reaction force, CoP: centre of pressure, ML: medio-lateral, AP: anterior-posterior, OG: overground, TM: treadmill, MoS: margin of stability, BoS: base of support, RoM: range of motion, EMG: electromyography, XCoM: extrapolated centre of mass.

## 4. Discussion

The purpose of this review was to collate and report recent methods of balance perturbation used during gait from available literature, with an additional focus on the outcome variables used to assess changes in gait. The most frequent method of balance perturbation was found to be the ML treadmill belt or treadmill translation, most commonly initiated at heel strike. Most included studies reported the use of spatiotemporal outcome variables, the most frequent outcome variable reported of which was step width. Some studies reported the collection of only spatiotemporal parameters, while other studies suggested that the addition of kinetic data in the outcome variables could have added depth to results or potentially provided an alternative viewpoint to the conclusion.

Studies perturbing participants via treadmill belt translation or floor translation during overground gait relocated participants’ BoS and CoP. Of these studies there was agreement regarding an increase in BoS, muscle activation, dynamic stability, and decreased CoM sway. There was disagreement on the results regarding MoS, joint angle RoM, step width and length, with some studies reporting increases and others reporting decreases. This discrepancy in the findings for outcome variables was likely a result of the heterogeneity in the choice of perturbation magnitude, gait phase of application, and the measurement approaches used to quantify gait variables. This may also have resulted in the differences in sample population ages, who may have responded differently to different perturbation types. Floor translation perturbations were also used to identify and investigate the concept of MoS and the relevant interactions and could be used to establish any links in participants’ spatiotemporal parameters of gait to dynamic stability [19,21,26,28]. As with spatiotemporal variables, the findings for MoS were not consistent.

All perturbations applied to participants CoM were via a waist belt attached to an external device worn during treadmill gait or by a physical therapist. These belts were attached to either cable pulleys [35,48], a pneumatic device [38], or a motor-driven device [36,37]. Within the results, there was agreement on increased muscle activation, dynamic stability, MoS, BoS peak vGRF, step time, and decreased CoP motion. There were conflicting results regarding step width and length, with some studies reporting increases and other decreases. While there were higher levels of agreement across studies that perturbed the CoM, there was significant heterogeneity in perturbation protocols and as a result of the reported outcomes.

Previous literature has stated that the most prevalent cause of the falls of OA participants residing in a long-term care facility was incorrect weight shifting, accounting for 41% of all recorded falls [49]. followed by trips and stumbles, causing 21% of falls, and just 3% of falls as a result of slipping. However, among younger adults, slipping is reported to be the most prevalent cause of falls [50]. A slip can be recreated in a laboratory setting by perturbation of the BoS. Both mechanisms have been captured in the reviewed approaches, and the diversity in the reported findings in this review highlight the importance of carefully selecting a relevant perturbation approach when attempting to estimate fall risk or model fall-causing paradigms.

The timing of the perturbation within the gait cycle can also affect results, and the most popular point in the gait cycle for perturbation initiation was a heel strike. Some studies did report perturbing participants at specific time intervals regardless of gait cycle and reported decreased CoP motion [38], decreased CoM sway [9], and decreased MoS and dynamic stability [22]. These results contradict previous literature as well as other studies in the review; while inconsistencies of methods are abundant, the timing of perturbation onset should not be discounted as having an influence on the results [21,26,35,51,52,53]. In addition to this, perturbation at heel strike was performed using split belt and single belt treadmills, providing contradicting motivation and intention of the perturbation. An AP perturbation of a single belt treadmill would reflect a two-footed simultaneous slip mechanism, whereas a split belt treadmill perturbation allows for researchers to investigate paradigms of a single foot slipping, which may be common at heel strike.

One study compared participant response to the same balance perturbation applied during overground and treadmill gait and found no significant difference between walking conditions for step length, step width, step time, and foot placement [37]. However, previous research has shown treadmill gait to be significantly less complex than overground gait, although the effect of reduced complexity is unlikely to be detectable with spatiotemporal variables alone [54].

Limitations: The research question was specific to recent perturbation methods; however, limiting the search to publications during or after 2015 may have excluded studies which would otherwise have justified inclusion. Due to the broad nature of the literature search and no exclusion criteria based on participant parameters, this may have predisposed the search results to an increased variety of perturbation methods due to differing populations, presenting a variety of ages, physical conditions, and fall history. However, this does allow the review to assess the scope of the perturbation methods currently in use. However, it is important that the implications of different perturbation types on results is understood to improve the interpretation and application of those findings.

While this review specifically focused on the kinetic and kinematic biomechanical outcomes of a perturbation, an up-to-date review on central and peripheral measurements of motor control would benefit this research area and would align outcomes in this field of study. In addition, as this field is developing rapidly, a technical overview of novel and new approaches of motor control (central and peripheral), kinetic and kinematic, with an eye on psychometric measurement properties, would be beneficial to the research community.

Clinical application: A recent meta-analysis has found that stepping tasks have a moderate accuracy in identifying fallers vs. non-fallers [55]. Active mechanical perturbation may provide an alternative, more accurate, and specific option for the identification of fallers. However, as the presented findings show, there is a lack of standardisation in the gait perturbation testing and training methods used to assess and improve balance recovery. The same findings also extend to the outcome variables used by researchers. For standardised testing to be developed, a set of outcome variables need to be identified that most accurately assess fall risk. Further, research on fall-specific perturbation methods is needed to enable the identification of fallers prior to the first fall based on laboratory data collection [10,56,57].

## 5. Conclusions

This study summarises perturbation methodologies and provides insight into the outcome variables used in assessing these methods among participants. While there are novel technological advances in this field of research, there is a lack of consideration of the selection of perturbation type and outcome variables selected and the impact these decisions have on the results of the study. This potentially limits the progress of the translation of research findings from the field into clinical settings.

## Appendix A

**Table A1:** Full risk of bias assessment.

Reference	1	2	3	4	5	6	7	8	9	10	11	12	13	14	15	16	17	18	19	20
Aprigliano et al. [20]	Y	Y	N	N	U	U	N	Y	Y	N	N	Y	U	N	Y	Y	Y	Y	N	Y
Aprigliano et al. [18]	Y	Y	N	Y	U	U	N	Y	Y	Y	Y	Y	U	N	Y	Y	Y	N	N	Y
Aprigliano et al. [19]	Y	Y	N	Y	U	U	N	Y	Y	Y	Y	Y	U	N	Y	Y	Y	N	N	Y
Arvin et al. [39]	Y	Y	N	Y	U	Y	N	Y	Y	Y	Y	Y	U	N	Y	Y	Y	Y	N	Y
Best et al. [40]	Y	Y	N	N	U	U	N	Y	Y	Y	Y	Y	U	N	Y	Y	Y	Y	N	Y
Capin et al. [47]	Y	Y	N	Y	U	U	N	Y	Y	Y	Y	Y	U	N	Y	Y	Y	Y	N	Y
Francis et al. [42]	Y	Y	N	N	U	U	N	Y	Y	Y	Y	Y	U	N	Y	Y	Y	Y	N	Y
Golyski et al. [24]	Y	Y	N	N	U	U	N	Y	Y	Y	Y	Y	U	N	Y	Y	Y	Y	N	Y
Haarman et al. [25]	Y	Y	N	Y	U	U	N	Y	Y	Y	Y	Y	U	N	Y	Y	Y	Y	N	Y
Hof and Duysens [38]	Y	Y	N	N	U	U	N	Y	Y	Y	Y	Y	U	N	Y	Y	Y	N	N	Y
Kao et al. [31]	Y	Y	N	N	U	U	N	Y	Y	Y	Y	Y	U	N	Y	Y	Y	Y	N	Y
Kreter et al. [41]	Y	Y	N	Y	Y	Y	N	Y	Y	Y	Y	Y	U	N	Y	Y	Y	Y	N	Y
Kurz et al. [9]	Y	Y	N	Y	Y	Y	N	Y	Y	Y	Y	Y	U	N	Y	Y	Y	Y	N	Y
Lee-Confer et al. [46]	Y	Y	N	Y	U	U	N	Y	Y	Y	Y	Y	U	N	Y	Y	Y	Y	N	Y
Madehkhaksar et al. [26]	Y	Y	Y	N	U	U	N	Y	Y	Y	Y	Y	U	N	Y	Y	Y	Y	N	Y
Martelli et al. [35]	Y	Y	N	N	U	U	N	Y	Y	Y	Y	Y	U	N	Y	Y	Y	Y	N	Y
Martelli et al. [7]	Y	Y	N	N	U	U	N	Y	Y	Y	Y	Y	U	N	Y	Y	Y	Y	N	Y
McIntosh et al. [43]	Y	Y	N	N	U	U	N	Y	Y	Y	Y	Y	U	N	Y	Y	Y	Y	N	Y
Nestico et al. [32]	Y	Y	N	Y	U	U	N	Y	Y	Y	Y	Y	U	N	Y	Y	Y	Y	N	Y
Onushko et al. [27]	Y	Y	N	N	U	U	N	Y	Y	Y	Y	Y	U	N	Y	Y	Y	Y	N	Y
Punt et al. [22]	Y	Y	N	Y	Y	Y	N	Y	Y	Y	Y	Y	U	N	Y	Y	Y	Y	N	Y
Rieger et al. [21]	Y	Y	N	N	Y	Y	N	Y	Y	Y	Y	Y	U	N	Y	Y	Y	Y	N	Y
Roeles et al. [28]	Y	Y	N	Y	U	Y	N	Y	Y	Y	Y	Y	U	N	Y	Y	Y	Y	N	Y
Rosenblum et al. [29]	Y	Y	N	Y	U	U	N	Y	Y	Y	Y	Y	U	N	Y	Y	Y	Y	N	Y
Rosenblum et al. [33]	Y	Y	Y	Y	U	U	N	Y	Y	Y	Y	Y	U	N	Y	Y	Y	Y	N	Y
Rutherford et al. [45]	Y	Y	N	Y	Y	Y	N	Y	Y	Y	Y	Y	U	N	Y	Y	Y	Y	N	Y
Sheehan et al. [30]	Y	Y	N	N	U	U	N	Y	Y	Y	Y	Y	U	N	Y	Y	Y	N	N	Y
Shulman et al. [44]	Y	Y	N	N	U	U	N	Y	Y	Y	Y	Y	U	N	Y	Y	Y	Y	N	Y
Taborri et al. [34]	Y	Y	N	Y	U	U	N	Y	Y	Y	Y	Y	U	N	Y	Y	Y	N	N	Y
van Hal et al. [23]	Y	Y	N	Y	U	U	N	Y	Y	Y	Y	Y	U	N	Y	Y	Y	N	N	Y
Vervoort et al. [8]	Y	Y	N	Y	U	U	N	Y	Y	Y	Y	U	U	N	U	U	U	U	N	Y
Vlutters et al. [36]	Y	Y	N	Y	U	U	N	Y	Y	Y	Y	Y	U	N	Y	Y	Y	N	N	Y
Zadravec et al., 2017 [37]	Y	Y	N	N	U	U	N	Y	Y	Y	Y	Y	U	N	Y	Y	Y	Y	N	Y

Y: yes, N: no, U: unspecified.
